# The effect of laparoscopic and abdominal surgery on the treatment of ectopic pregnancy: a systematic review and meta-analysis

**DOI:** 10.3389/fmed.2024.1400970

**Published:** 2024-08-27

**Authors:** Lin Zhai, Yinping Chen, Shengzhi Zhang

**Affiliations:** ^1^Department of Gynecology, Ningbo Medical Center Lihuili Hospital, Ningbo, Zhejiang, China; ^2^Department of Gynecology, Yuyao People's Hospital, Yuyao, Zhejiang, China

**Keywords:** laparoscopic surgery, abdominal surgery, ectopic pregnancy, meta-analysis, Cochrane RoB2.0

## Abstract

**Objective:**

This study aimed to compare the efficacy of laparoscopic surgery (LS) and abdominal surgery (AS) in ectopic pregnancy surgery.

**Methods:**

A computer-based search was conducted in several databases such as CNKI, Wanfang data, VIP data, Chinese Biomedical Literature Database (CBM disc), PubMed, Cochrane Library, Embase, and others to gather domestic and foreign literature on treating ectopic pregnancy. This search was carried out from the inception of each database to July 2022. The literature review was performed using Endnote X9 software, and the data were analyzed using STATA 15.1 software for the meta-analysis.

**Results:**

Eight articles that met the inclusion criteria were included in the study. The meta-analysis showed that the laparoscopic group had shorter operation time than AS group [SMD = −1.28, 95%CI (−2.02, 0.54), *p* = 0.001], had less intraoperative bleeding [SMD = −3.06, 95%CI (−3.82, −2.31), *p* < 0.01], shorter postoperative anus exhaust time [SMD = −2.60, 95%CI (−3.26, −1.93), *p* < 0.01], and shorter hospital stay [SMD = −1.74, 95%CI (−2.09, −1.39), *p* < 0.01] with few complications [RR = 0.22, 95%CI (0.08, 0.55), *p* = 0.001].

**Conclusion:**

LS has more evident advantages in the treatment of patients with ectopic pregnancy. However, due to the lack of English literature that meets the inclusion criteria, further studies are needed to determine if LS has the same efficacy for European and American populations.

## 1 Preface

Ectopic pregnancy (EP) refers to the implantation and development of a fertilized egg outside the uterine coelom, including well-known cases such as cervical pregnancy (1). As one of the prevalent types of acute abdomen in obstetrics and gynecology, the incidence of EP is significantly increasing, accounting for 2–3% of other pregnancy diseases (2). It has become one of the leading causes of maternal death in the first trimester (3, 4). Patients with EP who are found in time and have mild symptoms are usually treated conservatively by taking drugs such as methotrexate (5, 6). Sathyaprakash et al. (7) focussed on explainable AI models, federated learning approaches, and integration of real-time sensor data. Additionally, a meta-analysis comparing laparoscopic surgery (LS) and abdominal surgery (AS) in treating EP showed that LS had several advantages, including shorter operation times, less bleeding, shorter hospital stays, and fewer complications. However, further studies are needed to determine if the same efficacy applies to European and American populations. For patients with EP who meet the surgical indications, the surgical treatment usually used in the past is AS (8). Although many primary medical institutions still use this surgical procedure because laparoscopic techniques have been popularized in recent years because of their characteristics of minor trauma, rapid recovery, and insignificant scars, the advantages of gynecological surgery are gradually highlighted, which has become one of the most commonly used surgical procedures for the treatment of EP and is favored by patients and doctors (9). However, LS is not operated under direct vision but requires operation through electrosurgical equipment under two-dimensional images, often requiring Trendelenburg position. Some complications are not easily detected during surgery, and these complications can be challenging to manage and often require completion by an experienced surgeon (AS). Dr. P.M. Kumar et al. proposed that wind and solar energy can generate electricity. Their dilute nature poses challenges that need to be addressed using solar panels and expert systems such as artificial neural network-based expert systems. According to a meta-analysis of eight articles, LS is more effective than AS in treating EP (10). In addition, LS is more costly than AS, and electrocoagulation hemostasis carries a risk of damaging the fallopian tubes and ovaries. Determining the costs associated with evaluated interventions or procedures is crucial in research studies. Researchers can obtain cost data from hospital billing records, healthcare databases, or patient surveys. Standardized cost estimation methods or detailed cost analysis can be used to ensure accuracy and reliability. Careful attention to detail, transparent reporting of methods, and thorough documentation of all expenses incurred are necessary (11). Although AS involves a large surgical incision, it also involves a broad surgical field. It can quickly deal with the complications that occur during surgery, which is an irreplaceable advantage of LS (12). More and more researchers have carried out studies on the clinical efficacy of the two surgeries, which are being evaluated through randomized controlled trials, but most of them have small sample sizes (13). EP, which is the implantation of the fertilized ovum outside the uterine cavity, is a significant cause of morbidity and mortality among women of reproductive age. Surgical intervention is crucial in managing an EP. There is an ongoing debate about the best surgical approach regarding effectiveness, safety, and postoperative outcomes. This systematic review and meta-analysis aim to clarify the comparative effectiveness of laparoscopic and AS in treating EP (14, 15). The study’s objective is to conduct a meta-analysis to consolidate and compare the impact of each study, comprehensively assess the clinical effectiveness of the two surgeries, and offer pertinent insights and theoretical guidance for future practical application in a clinical setting.

## 2 Materials and methods

### 2.1 Search strategy

Two authors conducted a computer search of various domestic and international databases, including China National Knowledge Infrastructure, Wanfang Data, VIP Data, Chinese Biomedical Literature Database (CBM disc), PubMed, Cochrane Library, and Embase. They used search terms such as “laparoscopic surgery,” “laparotomy,” and “ectopic pregnancy,” along with subject headings, and also reviewed references to identify additional relevant clinical trials meeting the inclusion criteria.

### 2.2 Literature screening procedure

Two researchers checked the literature using Endnote X9 software, performed preliminary screening by reading titles and abstracts, proposed literature journals not related to the subject matter, downloaded the full text and read the full text for judgment, proofread the articles with objections by a third researcher, and studied and discussed whether three researchers included them. Data extraction includes ① authors of literature, basic information such as publication year; ② intervention measures and sample size of the test and control groups; and ③ conclusion of each clinical study.

### 2.3 Criteria for inclusion in the study

(1) A medical study that looks at how well LS works compared to AS in treating EP patients.
(2) There is a definite diagnosis of EP before the operation, at least one outcome indicator;
(3) Detailed original data, no difference in general conditions;
(4) All sample studies were approved by the ethics committee.

Exclusion criteria:

(1) Exclude cohort study, case–control study, and other non-randomized controlled trials;
(2) Exclude meeting and individual case reports;
(3) Exclude animal tests;
(4) Exclude articles with repeated data and retain the latest studies.

### 2.4 Literature bias risk assessment

Cochrane RoB2.0 was used to assess the risk of publication bias in the literature. It was evaluated based on the following six aspects: ①bias during randomization; ②bias away from established interventions; ③bias in outcome measurements; ④bias in missing outcome data; ⑤bias in selective reporting of results; and ⑥overall bias. It was performed independently and cross-checked by two inspectors.

### 2.5 Statistical analysis

Forest plots generated with STATA 15.1 software for descriptive purposes. Egger’s test was used to assess publication bias and conduct sensitivity analysis to ensure the stability of the combined effect size. A statistical significance level of *p* < 0.05 was used, and the relative risk (RR), along with a 95% confidence interval (95% CI), was utilized to quantify complications. In contrast, variables used for efficacy analysis in measurement data included a 95% CI and standardized mean difference (SMD). Heterogeneity was quantitatively assessed using I2. Suppose no significant heterogeneity was found (I2 < 50%), meta-analysis was conducted using the fixed-effect model, followed by sensitivity analysis to investigate sources of heterogeneity. In cases where significant clinical heterogeneity was identified, meta-analysis was conducted using the random-effect model after excluding heterogeneity sources.

## 3 Results

### 3.1 Literature search results

**Figure 1:**
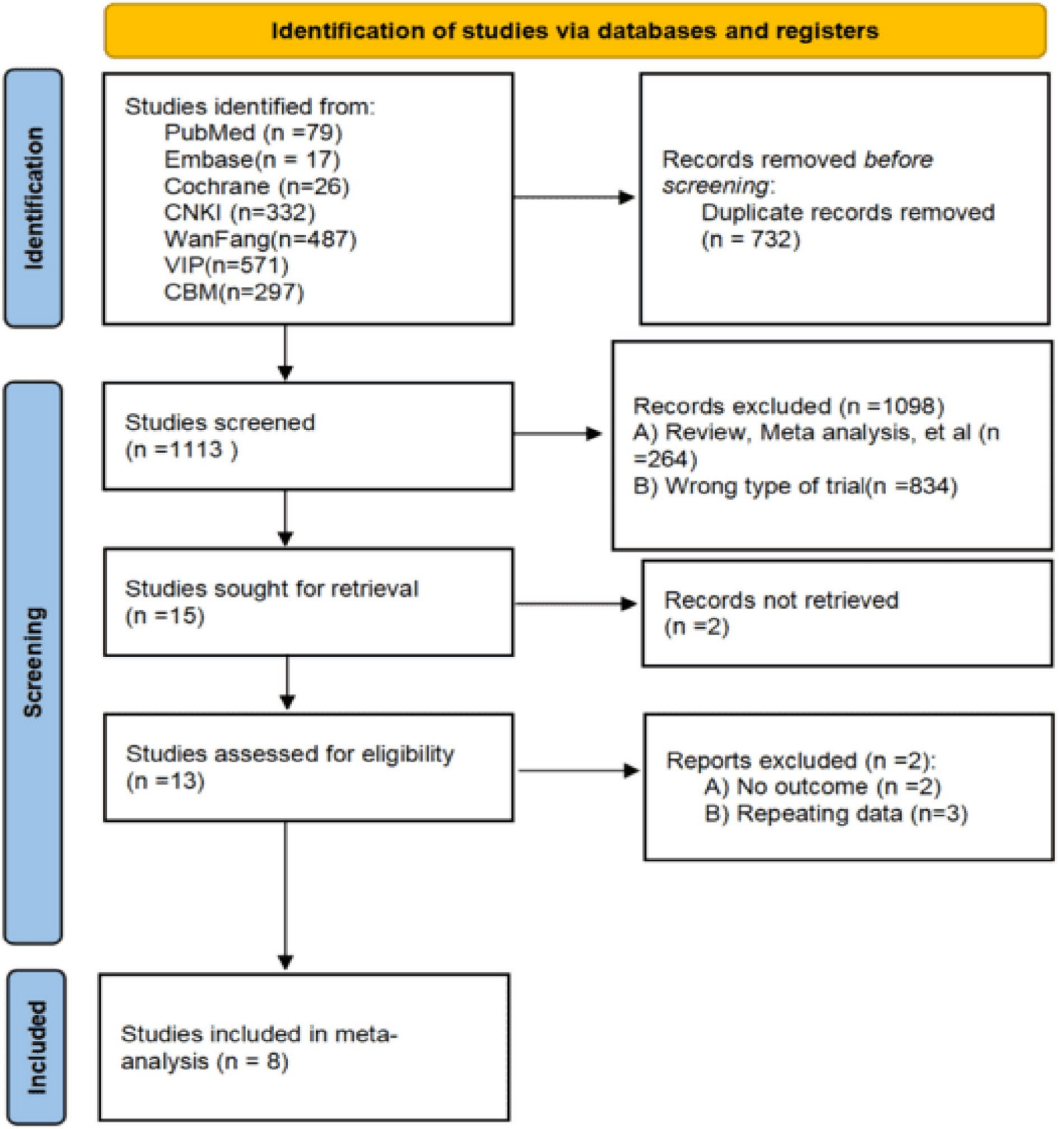
Literature screening flowchart.

Eight studies (1,006 patients with EP) were finally included among seven databases after strictly following the literature screening process and accurately checking the inclusion and exclusion criteria. The literature screening flowchart is depicted in Figure 1, while the basic information about the literature is presented in Table 1.

**Table 1:** Basic characteristics of the literature.

Study	Year	Age		Total		Interventions		Outcome measurements
		*T*	*C*	*T*	*C*	*T*	*C*	
Jinping Z (6)	2018	29.01 ± 3.54	28.74 ± 3.43	40	40	LS	AS	①②③④⑤
Sathyaprakash P (7)	2012	22.65 ± 6.50	26.33 ± 6.82	98	98	LS	AS	①②③④⑤
Haiying L (8)	2016	26.35 ± 3.17	–	75	75	LS	AS	①②③
Xiuping Z. A (9)	2010	–	–	43	43	LS	AS	①②③
Kumar PM (10)	2013	27.2 ± 5.9	26.9 ± 7.1	124	124	LS	AS	①②③④⑤
Guiping G (11)	2021	31.2 ± 3.8	31.5 ± 3.9	33	33	LS	AS	①②③④
Peng J (12)	2020	25.30 ± 4.60	2.42 ± 4.50	30	30	LS	AS	①②③④
Deli Z (13)	2010	31.36 ± 5.46	27.18 ± 4.02	60	60	LS	AS	①②③④

*T, experimental group; C, control group; LS, laparoscopic surgery; AS, abdominal surgery; ①operation time; ②intraoperative blood loss; ③anus exhaust time; ④hospital stay; ⑤complications.*

### 3.2 Risk of bias and assessment for included studies’ results

**Figure 2:**
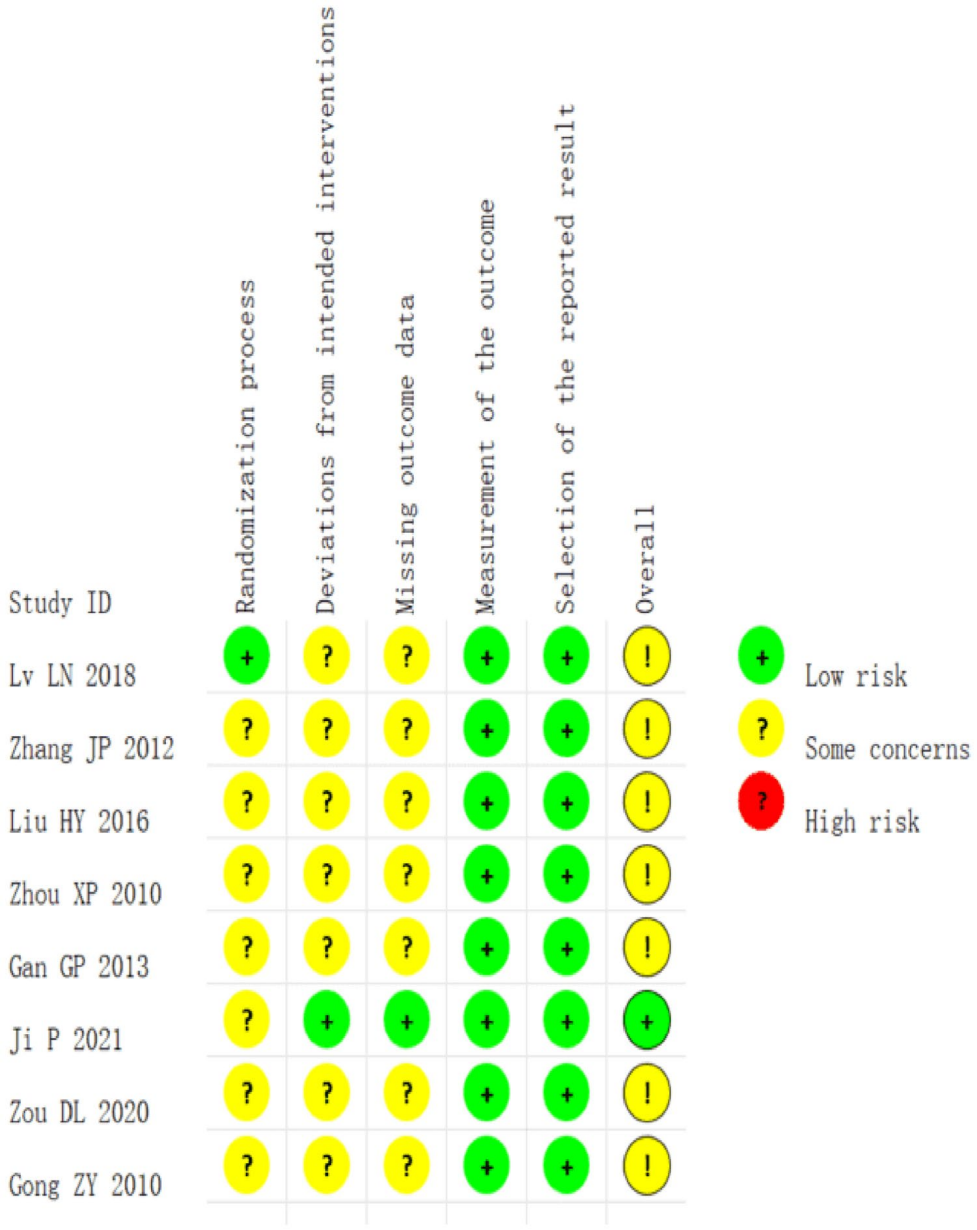
Detailed plot of bias of eight included literature: based on Cochrane RoB 2.0.

**Figure 3:**
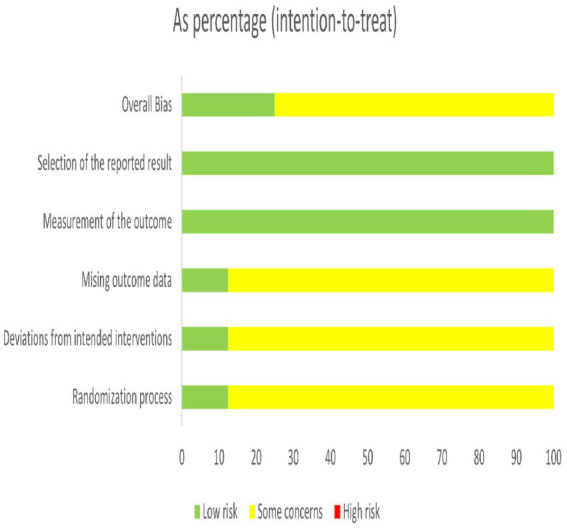
Summary plot of bias for the eight included literature: based on Cochrane RoB 2.0.

All the included studies were randomized controlled trials. Among them, one study used a good randomization method, one reported the double-masked method with the highest quality, and the rest did not mention the blind method or other allocation concealment. There were no cases of missing outcome data or selective bias, as shown in Figures 2, 3.

### 3.3 Operative time

**Figure 4:**
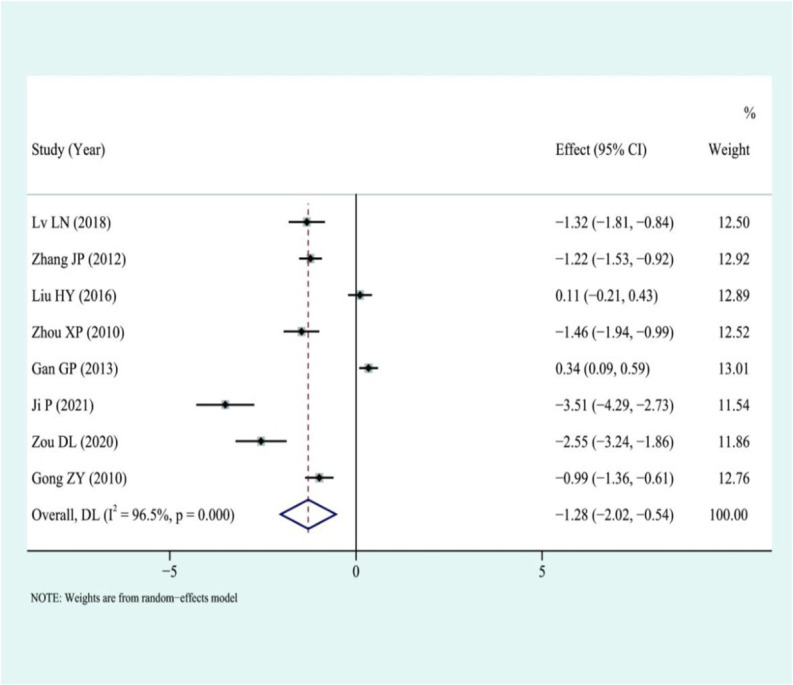
Forest plot for combined operative time results.

Eight studies were included, and they showed that AS required longer operative times than laparoscopy, I2 = 96.5%, using a random-effects model: SMD of −1.28 with a 95% CI of (−2.02, 0.54) was statistically significant with a *p*-value of 0.001. This indicates that the discrepancy was significant statistically, as shown in Figure 4.

### 3.4 Intraoperative bleeding loss

**Figure 5:**
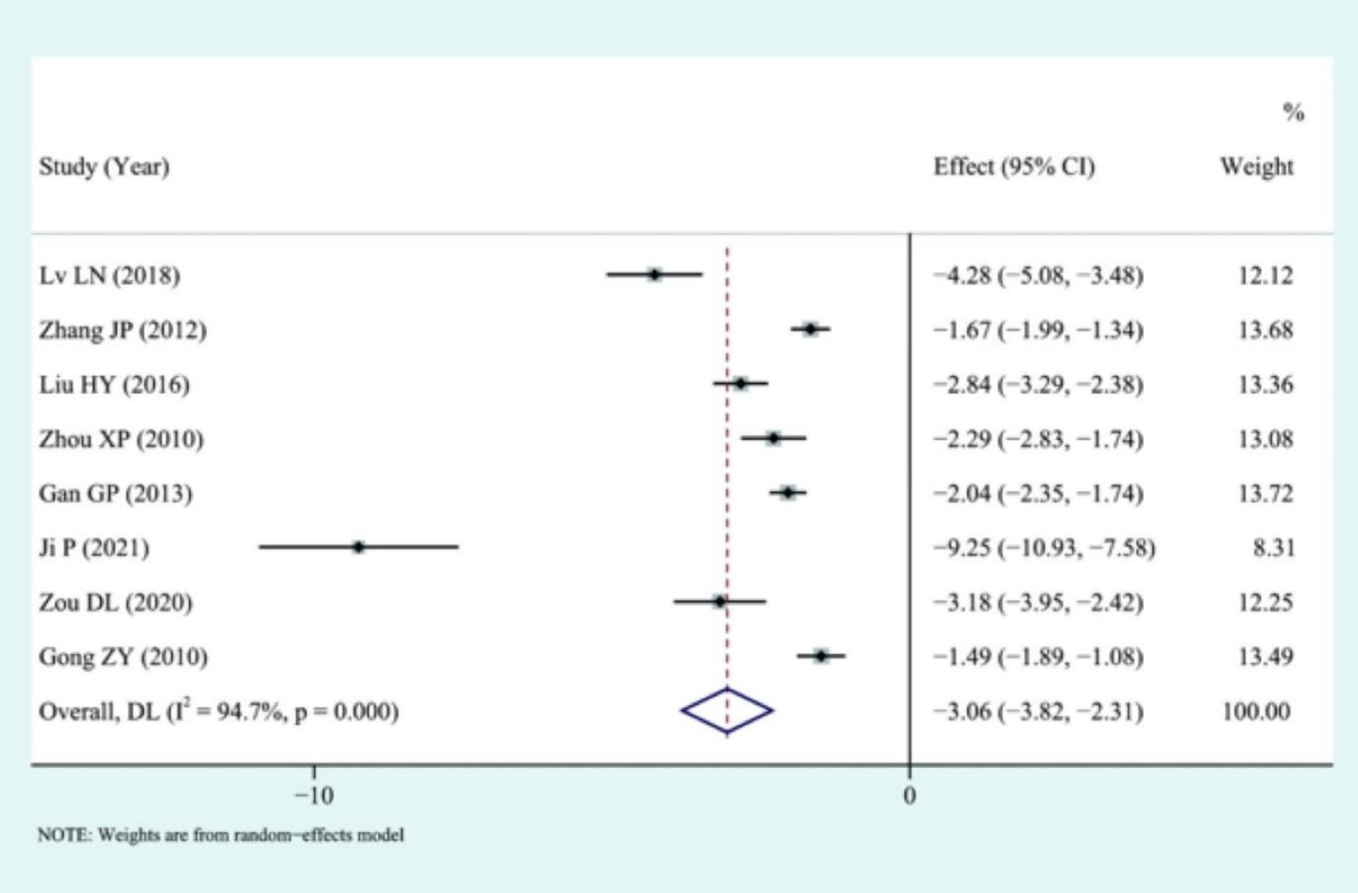
Forest plot of intraoperative blood loss results.

All studies compared intraoperative blood loss, and the analysis showed that LS could significantly reduce intraoperative blood loss (I2 = 94.7%) using a random-effects model: [SMD = −3.06, 95%CI (−3.82, −2.31), *p* < 0.01], the results suggested that there was statistical significance, as illustrated in Figure 5.

### 3.5 Anus exhaust time

**Figure 6:**
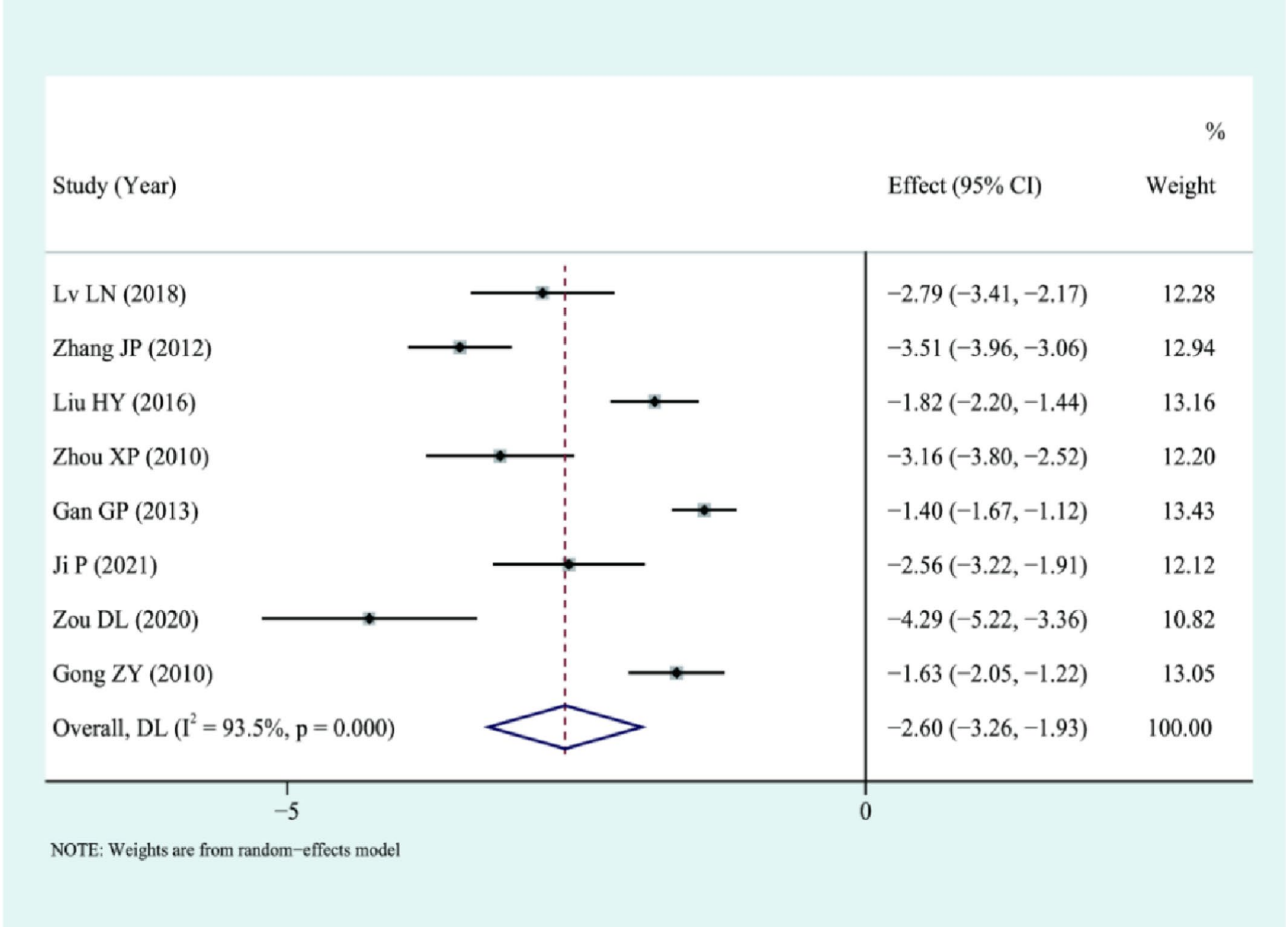
Forest plot for combined results of anus exhaust time.

Eight studies compared the time to the first postoperative flatus. The analysis showed that LS could reduce the time to first postoperative flatus, suggesting a shorter time to recover bowel function, I2 = 93.5%, using a random-effects model: [SMD = −2.60, 95%CI(−3.26, −1.93), *p* < 0.01], suggesting that the results were statistically significant, as illustrated in Figure 6.

### 3.6 Hospital stay

**Figure 7:**
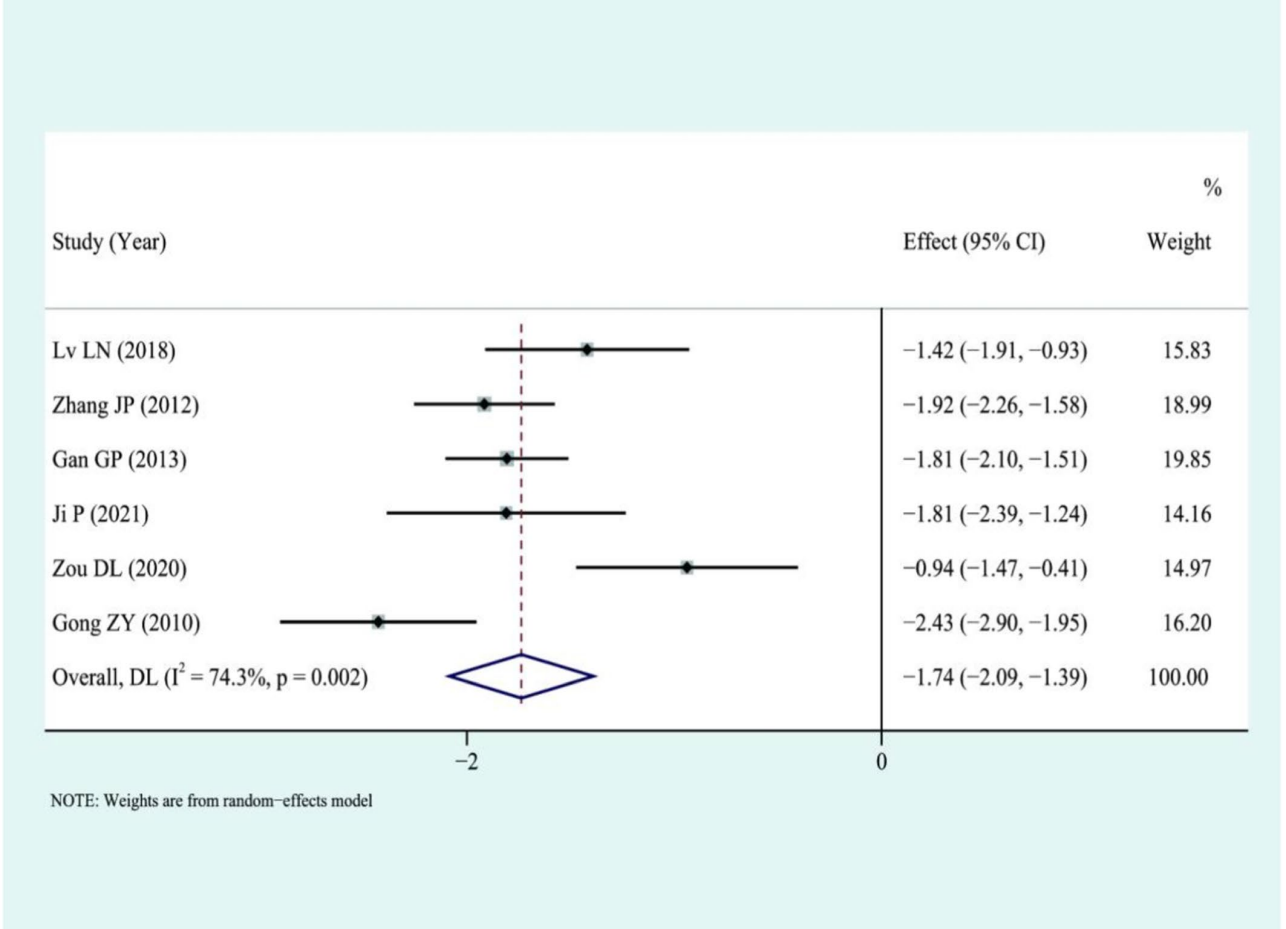
Forest plot for combined results of hospital stay.

Six studies compared the length of hospital stay. The analysis showed that LS could significantly shorten the length of hospital stay compared to traditional laparotomy (I2 = 74.3%) using a random-effects model: [SMD = −1.74, 95%CI (−2.09, −1.39), *p* < 0.01]. The results were deemed statistically significant, as illustrated in Figure 7.

### 3.7 Complications

**Figure 8:**
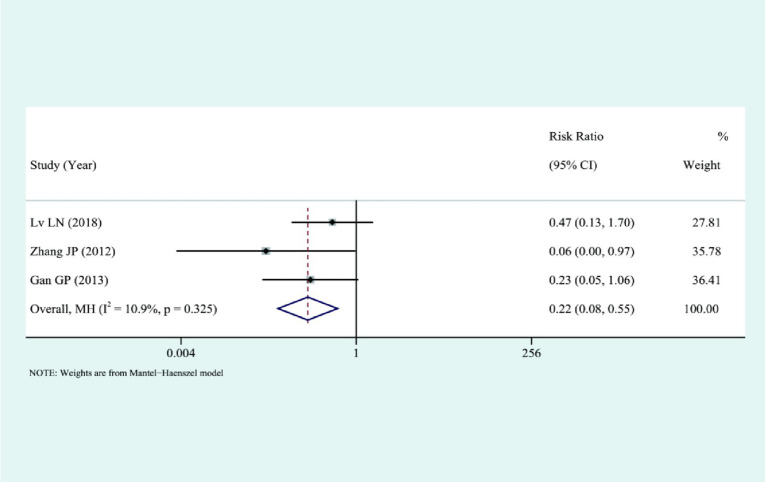
Combined forest plot of complication results.

Three studies compared the complications among the two sets, and the results presented that there were significantly fewer complications in the laparoscopic group compared to AS, I2 = 10.9%, fixed-effect model was selected: [RR = 0.22, 95%CI (0.08, 0.55), *p* = 0.001], and the difference was statistically significant, as shown in Figure 8.

### 3.8 Publication bias testing

**Figure 9:**
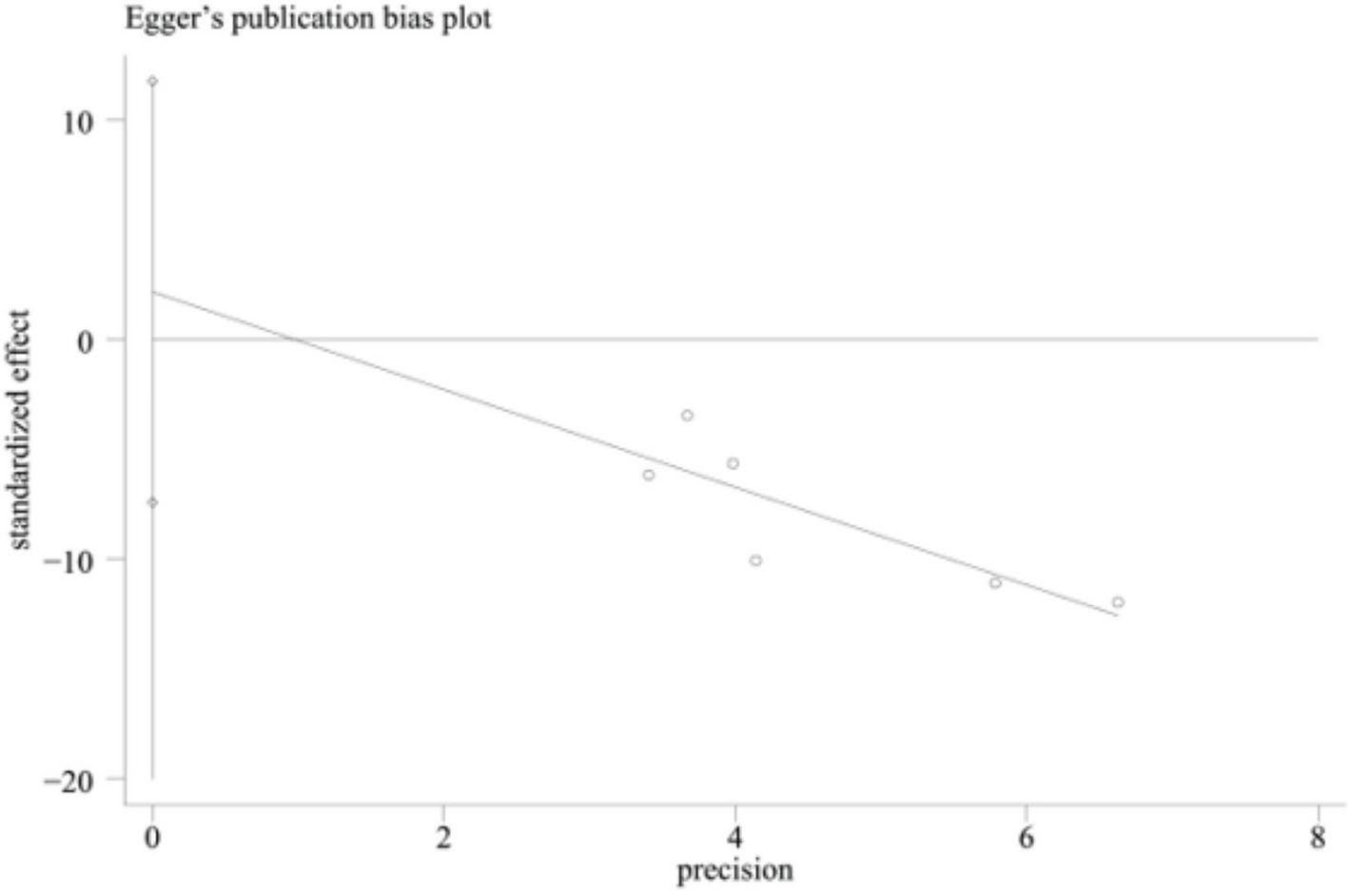
Egger’s test.

Egger’s test was performed for the duration of hospitalization, and the result was *p* = 0.556. The analysis showed no significant publication bias, as illustrated in Figure 9.

### 3.9 Sensitivity analysis

**Figure 10:**
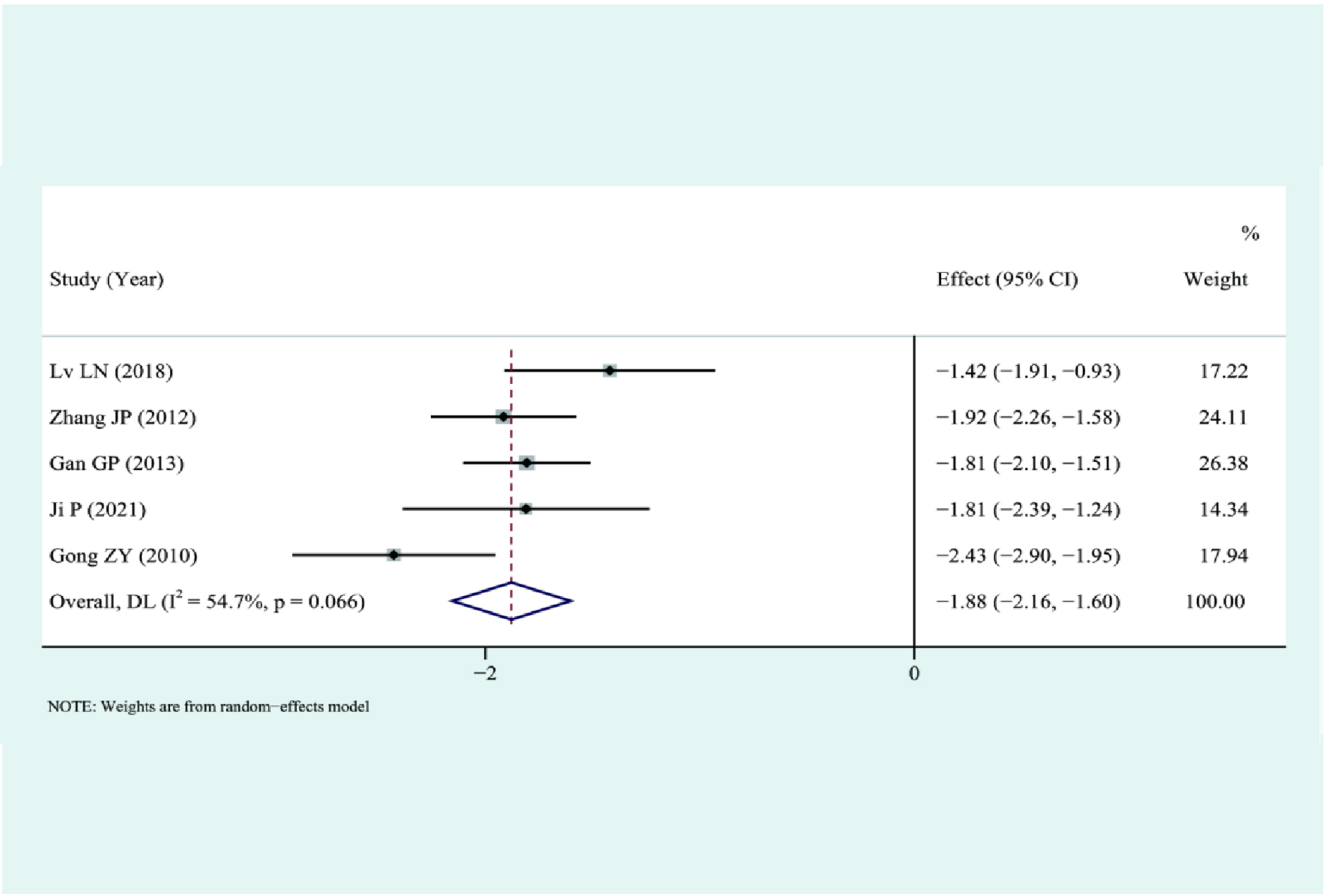
Sensitivity analysis.

**Figure 11:**
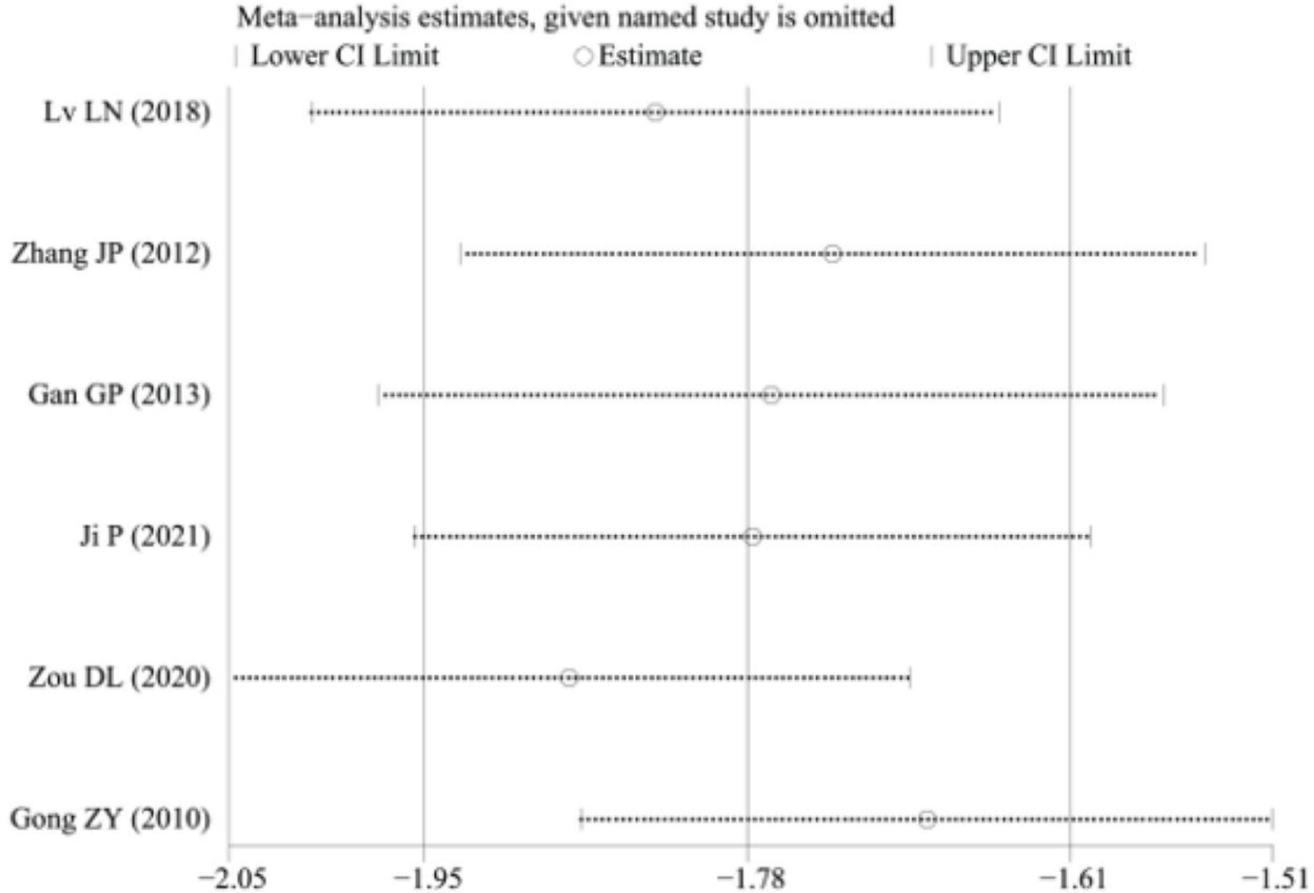
Length of hospital stay (sensitivity analysis).

A sensitivity analysis was conducted to determine the length of hospital stay, as illustrated in Figure 10. The results indicated that the heterogeneity may have originated from the study by Peng J. (12). After excluding this study, a meta-analysis revealed an I2 value of 54.7%. Due to specific clinical heterogeneity in the trial, a random-effects model was chosen, yielding an SMD of −1.88 with a 95% CI of −2.16 to −1.60 and a *p*-value of less than 0.001. Results indicate a statistically significant difference in Figure 11.

## 4 Discussion

There are many types of EP, including tubal pregnancy, ovarian pregnancy, abdominal pregnancy, and broad ligament pregnancy, of which approximately 90% are tubal pregnancy (16). In tubal pregnancy, the ampulla of the water is the most common, followed by the isthmus and fimbria. Although interstitial pregnancy is the least common, its symptoms are the most serious. EP is common and frequent in gynecological emergency departments, pelvic inflammatory disease history, EP history, tubal injury, and surgical history are common causes of EP (17), mainly related to the following factors: ① family planning: prevention of contraceptive devices will change the intrauterine environment and increase the possibility of EP; the use of compound oral contraceptives, especially low-dose pure progestogen contraceptives, can make tubal peristalsis disorder, increase the chance of tubal pregnancy; Recanalization and fistula formation occur after tubal sterilization, an EP occurs when sperm enters the fallopian tube through the fistula; induced abortion, mid-term pregnancy abortion, and medical abortion can easily cause EP; ② various sexually transmitted diseases can cause endocervical mucositis, endometritis, and salpingitis, and ultimately lead to infertility and tubal pregnancy; ③ other factors: such as luteal phase dysfunction, premature sexual intercourse, smoking, and multiple sexual partners are also high-risk factors for Junchao Q, et al. (18) in recent years, there has been a significant increase in the incidence of EP both domestically and internationally, making it the primary cause of maternal mortality during early pregnancy. Due to the current development of serum β-HCG detection technology and the continuous progress of gynecological ultrasound diagnostic techniques, especially laparoscopic techniques, which have been widely used, the vast majority of patients with EP can be diagnosed and treated in time at its early stage (19), thus reducing the incidence of death.

The management of EP involves both surgical and medical interventions. Conservative treatment is mainly performed with methotrexate (20), and EP patients who fail to respond to medical treatment or are at risk of massive bleeding should undergo prompt surgical treatment. In the past, EP surgery usually required laparotomy. However, this approach was invasive for the patient and necessitated waiting for a precise clinical diagnosis and clear surgical indications before proceeding with the surgery. With the development of laparoscopic techniques, LS can not only help patients to make early diagnoses but also perform surgical treatment with the support of necessary conditions for decisive hemostasis to avoid stenosis at the corresponding site of the fallopian tube caused by suture hemostasis during laparotomy (21).

Pittaway et al. (22) investigated the predictors of pain development after laparoscopic adnexectomy. They also conducted a systematic review and meta-analysis comparing the efficacy of laparoscopic surgery and abdominal surgery in the treatment of EP. They discussed the pathophysiology of surgical postoperative pain, the different macro-factors contributing to it, and the benefits of tailored multimodal analgesic strategies. The systematic review and meta-analysis found that LS is more effective than abdominal approaches in managing EP. The study by Wood, Carl et al.’s (23) aimed to compare laparoscopic adnexectomy with conventional laparotomy. A comparison was made between a group of 26 patients who had adnexectomy through laparotomy in the past and a group of 64 patients who underwent laparoscopic adnexectomy in the present. The findings indicated that laparoscopic adnexectomy provides significant benefits, including reduced surgery duration, decreased blood loss, shorter hospitalization, lower expenses, and quicker recovery. No disparities were noted in substantial complications, blood transfusions, adhesion formation, or the percentage of women reporting relief of pain symptoms.

Restaino et al. (24) research article “Laparoscopic Adnexectomy: Indications, Technique, and Results” was published in the Australian and New Zealand Journal of Obstetrics and Gynaecology in 1992. According to the article, 38 patients underwent laparoscopic adnexectomy with minimal blood loss, and all but two patients were discharged within 24 h of the surgery. The article suggests that laparoscopic adnexectomy is a preferable alternative to laparotomy due to reduced risks and faster recovery. According to Restaino et al. (25) ectopic pregnancies are rare and can be life-threatening, occurring in 1–2% of all pregnancies. It is even rarer to have an EP on the same side as a previous tubal removal after salpingectomy, which calls for multidisciplinary management.

According to Restaino et al. (26) interstitial pregnancy occurs when the embryo implants in the interstitial section of the Fallopian tube, which is a rare type of EP. Diagnosis can be made through transvaginal ultrasound; treatment options may include medical or surgical approaches. Surgical options such as laparoscopic unilateral colostomy or unilateral salpingectomy may be considered if medical treatment fails. When deciding on the correct treatment, it is essential to consider factors related to fertility. A 41-year-old woman encountered a complicated EP following the use of emergency contraception containing ulipristal acetate. She had a laparoscopic salpingectomy with no issues. This is the first documented instance of a highly developed EP in a woman who utilized emergency contraception containing ulipristal acetate.

This systematic review found that compared to the laparotomy group, the laparoscope group experienced shorter operation times, reduced intraoperative blood loss and hospital stays, earlier recovery, and a lower incidence of complications. This suggests that LS is a less invasive and safer option for treating EP. The shorter operation times for the laparoscope group can be attributed to the simplicity of operating the laparoscope and the fewer wounds for patients. This ultimately saves time on disinfection and suturing and allows for more precise management of the surgical site. The intraoperative blood loss was because the incision was smaller during laparoscopic operation, and monopolar and bipolar electrocoagulation could stop bleeding even on the surgical incision and avoid excessive bleeding. The postoperative exhaust time showed that the laparoscopic group was earlier, which may be related to the patient’s physical recovery, and the laparoscopic group had faster recovery due to minor intestinal function injury. The duration of hospitalization was presumably due to the patient’s more stable vital signs. The laparoscopic group was also superior in terms of complications, which reflected the advantages of the laparoscopic group regarding safety.

The limitations of this study include:

(1) All included articles are Chinese, and whether the conclusions apply to European and American populations remains to be studied;
(2) The method of randomization in the literature is unclear, and the blind method is not used well;
(3) The duration of abdominal pain, the duration of an indwelling urinary catheter, and other indicators are not evaluated.

## 5 Conclusion

In summary, the study findings exceptionally endorse laparoscopy as the top choice for managing EP because of its many advantages, such as decreased risk, quicker healing, and shorter hospitalization. By integrating findings from various sources on a local and global scale, the study indicates that patients who undergo LS have more favorable results than those who undergo AS. In particular, the laparoscopy group demonstrated benefits in terms of time to postoperative recovery, length of surgery, duration of hospital stays, amount of blood loss, and rates of complications. Overall, these results highlight the superiority and benefits of LS compared to AS in treating EP. They emphasize the significance of thorough evaluations of complications and reproductive results after various surgical methods. Moreover, additional randomized controlled trials are needed to improve the evidence backing the effectiveness of LS for EP, providing possibilities for improving patient care, treatment results, and surgical practices.

## Data availability statement

The original contributions presented in the study are included in the article/supplementary material, further inquiries can be directed to the corresponding author.

## Author contributions

LZ: Conceptualization, Writing – original draft. YC: Data curation, Writing – original draft. SZ: Formal analysis, Writing – original draft.
